# Cyclooxygenase/lipoxygenase shunting lowers the anti-cancer effect of cyclooxygenase-2 inhibition in colorectal cancer cells

**DOI:** 10.1186/1477-7819-10-200

**Published:** 2012-09-26

**Authors:** Radhakrishnan Ganesh, Daniel JB Marks, Kevin Sales, Marc C Winslet, Alexander M Seifalian

**Affiliations:** 1 Division of Surgery and Interventional Science, University College London, Rowland Hill Street, London NW3 2PF, UK; 2 Centre for Molecular Medicine, University College London, 5 University Street, London WC1E 6JF, UK

**Keywords:** Apoptosis, Colorectal cancer, Cyclooxygenase, Eicosanoids, Lipoxygenase

## Abstract

**Background:**

Arachidonic acid metabolite, generated by cyclooxygenase (COX), is implicated in the colorectal cancer (CRC) pathogenesis. Inhibiting COX may therefore have anti-carcinogenic effects. Results from use of non-steroidal anti-inflammatory drugs inhibiting only COX have been conflicting. It has been postulated that this might result from the shunting of arachidonic acid metabolism to the 5-lipoxygenase (5-LOX) pathway. Cancer cell viability is promoted by 5-LOX through several mechanisms that are similar to those of cyclooxygenase-2 (COX-2). Expression of 5-LOX is upregulated in colorectal adenoma and cancer. The aim of this study was to investigate the shunting of arachidonic acid metabolism to the 5-LOX pathway by cyclooxygenase inhibition and to determine if this process antagonizes the anti-cancer effect in colorectal cancer cells.

**Methods:**

Three colorectal cancer cell lines (HCA7, HT-29 & LoVo) expressing 5-LOX and different levels of COX-2 expression were used. The effects of aspirin (a non-selective COX inhibitor) and rofecoxib (COX-2 selective) on prostaglandin E_2_ (PGE_2_) and leukotriene B_4_ (LTB_4_) secretion were quantified by ELISA. Proliferation and viability were studied by quantifying double-stranded DNA (dsDNA) content and metabolic activity. Apoptosis was determined by annexin V and propidium iodide staining using confocal microscopy, and caspase-3/7 activity by fluorescent substrate assay.

**Results:**

COX inhibitors suppressed PGE_2_ production but enhanced LTB_4_ secretion in COX-2 expressing cell lines (*P* <0.001). The level of COX-2 expression in colorectal cancer cells did not significantly influence the anti-proliferative and pro-apoptotic effects of COX inhibitors due to the shunting mechanism.

**Conclusions:**

This study provides evidence of shunting between COX and 5-LOX pathways in the presence of unilateral inhibition, and may explain the conflicting anti-carcinogenic effects reported with use of COX inhibitors.

## Background

Colorectal cancer (CRC) remains a leading cause of cancer death, with highest incidence in westernized populations. The pathogenic sequence is well-understood, with characteristic genetic and biochemical abnormalities underlying the adenoma-carcinoma progression [1]. The long phase of progressive premalignant lesions, coupled with the availability of appropriate investigations, provides an opportunity for intervention and primary prevention. A number of chemopreventative strategies have been postulated. There is substantial evidence that diet modifies risk, [2,3] and oral agents that show promise include folate, statins, calcium, ursodeoxycholic acid and cyclooxygenase (COX) inhibitors [4-7].

Metabolites of arachidonic acid (AA) are important mediators in the adenoma-carcinoma sequence [8-10]. COX and 5-lipoxygenase (5-LOX) are the key enzymes involved in the generation of prostaglandins and leukotrienes respectively from this precursor. These were originally identified as playing important roles in the modulation of inflammation. Cyclooxygenase has two isoforms: COX-1 and COX-2. The former is constitutively expressed in most tissues, whereas the latter is an immediate-to-early response gene [11]. It is undetectable in most normal tissues, but is upregulated in colorectal neoplasms and their precursor lesions, [12] in which levels of downstream prostaglandin E_2_ (PGE_2_) are also elevated [13]. Genetic manipulation studies have shown a causal role for COX-2 in carcinogenesis in cytological and animal models [14-16]. Inhibition of COX-2 activity reverses CRC carcinogenesis in these systems, [17] and has been shown to induce apoptosis, and inhibit proliferation and angiogenesis [18,19]. Similar data have also recently emerged for 5-LOX [20,21]. Expression of 5-LOX has been demonstrated in some cancer cells and is involved in the pathogenesis of cancer. Interestingly, 5-LOX appears to have similar mechanisms to COX-2 in the regulation of cell viability, although these two enzymes often utilize different signaling pathways. Furthermore, it was suggested that arachidonic acid might be shunted from one pathway to the other when a particular pathway is inhibited in the cellular processes of cancer [22] and inflammation [23].

COX inhibitors are potentially attractive drugs for the chemoprevention of colorectal cancer, and have been reported to induce regression of polyps in patients with familial adenomatous polyposis [24]. Observational studies suggest a protective effect of non-steroidal anti-inflammatory drugs (NSAIDs), [25] which non-specifically inhibit COX-2 and its isoform COX-1. Use of these agents is, however, unfortunately limited by gastrointestinal and renal side-effects [26]. Specific COX-2 inhibitors were developed to circumvent these issues, although reports suggest that they are associated with significant cardiovascular adverse effects [27].

Studies evaluating anti-carcinogenic properties of COX-2 inhibitors however have not shown consistent results. There is a mismatch between the growth-suppressing effect of COX-2 inhibitors [28,29] and pro-carcinogenic effect of prostaglandins [30]. It is possible that the shunting of AA between COX-2 and 5-LOX that utilize AA will bypass COX-2 inhibition. In cells expressing both enzymes, inhibition of one in isolation may shunt metabolism preferentially down the other pathway, leading to paradoxically increased production of selected eicosanoids. In the presence of COX-2 and 5-LOX expression in cancer cells, combined inhibition of these pathways would likely to be a more effective anti-cancer modality with fewer side-effects.

Here we investigated the effects of NSAIDs on eicosanoid production from colorectal cancer cell lines expressing different level of COX-2 and its consequent anti-neoplastic effects. In this study, we hypothesized that in the presence of shunting between COX-2 and 5-LOX pathway, COX-2 inhibition might fail to show anti-cancer effect irrespective of COX-2 expression. Thus shunting of AA between COX-2 and 5-LOX pathways that utilize AA may bypass COX-2 inhibition.

## Methods

### Cell lines

Three human colon adenocarcinoma cell lines (HCA-7, HT29 and LoVo) were studied *in vitro* (European Collection of Cell Cultures, Salisbury, UK). HCA-7 expresses functional COX-2, and was cultured in Dulbecco's Modified Eagle Medium (DMEM) with L-glutamine (Sigma, Gillingham, UK). HT29 expresses an enzymatically inactive COX-2 isoform [31], and was cultured in McCoy’s 5A medium (Sigma). LoVo is derived from a metastatic adenocarcinoma not expressing COX-2, and was grown in Ham’s F-12 medium with L-glutamine (Sigma). Media were supplemented with 10% fetal bovine serum, penicillin and streptomycin (2%). Cells were incubated at 37°C in 5% CO_2_, grown to 90% confluence in 75 cm^2^ flasks, and trypsinized and plated for experiments as described below.

Following overnight incubation, medium was exchanged for that containing test reagent. Rofecoxib (Merck, Nottingham, UK) was dissolved in DMSO to a stock concentration of 100 mM and was then diluted in medium to the final concentrations. Aspirin (Sigma) was dissolved in 1 M Tris-HCl to a stock concentration of 1 M with pH adjusted to 7. Negative controls were the equivalent media containing no drug.

### Eicosanoid production

PGE_2_ and leukotriene B_4_ (LTB_4_) secretion were quantified by ELISA (Cayman Chemicals, Tallinn, Estonia) as previously described [32]. Cells (1x10^5^) were plated overnight in 25 cm^2^ flasks and were then treated with the test reagents for 4 hours in serum-free medium. Supernatants were assayed and concentrations normalized to the number of adherent cells in the sample culture.

### Proliferation

Cells were grown in 25 cm^2^ flasks at a concentration of 1x10^5^ cells/ml, treated for 24, 48 or 72 hours, and were then trypsinized and centrifuged into pellets. These were homogenized using a 25 gauge needle to release DNA, which was measured using the Picogreen^TM^; dsDNA Assay kit (Invitrogen, Paisley, UK) as previously described [33]. We used this assay to quantify double-stranded DNA using a fluorescence readout (excitation: 485 nm, emission: 538 nm) on a Fluroskan Ascent FL spectrofluorometer (Thermo Life Sciences, Basingstoke, UK). Reduction in proliferation is expressed as a percentage of the appropriate control.

### Viability

Viability was assessed by alamar blue (Serotec, Oxfordshire, UK) reduction, which provides a colorimetric readout of the reducing environment of proliferating cells. Cells were plated in 24-well plates at a concentration of 1x10^4^ cells/ml, and treated for 24, 48 or 72 hours; viability at baseline was >95%. After treatment, medium was exchanged for that containing 10% alamar blue. After 4 hours, 100 μL of supernatant was transferred to a 96-well plate and read in a fluorescence plate reader (excitation: 560 nm, emission: 590 nm). Viability following drug treatment was compared to the appropriate control and expressed as a percentage.

### Apoptosis

Apoptosis was quantified by annexin-V and propidium iodide staining, using Annexin V-FITC apoptosis detection assay kit (Calbiochem, Nottingham, UK). Cells were plated in six-well plates at a concentration of 1x10^5^ cells/ml, and treated for 24, 48 and 72 hours, washed with phosphate-buffered saline (PBS) and treated with assay buffer, Annexin-FITC and propidium iodide as per the protocol described by the manufacturer. Apoptotic cells were detected under fluorescence microscopy, with early apoptotic cells exposing phosphatidyl serine at the cell wall and appearing green on the cell membrane surface and late apoptotic cells staining red throughout the cytoplasm. Apoptosis in each group was quantified as the percentage of apoptotic cells per high power field.

### Caspase-3/7 activity

The enzymatic activity of caspase-3/7 was measured using a commercially available fluorescent assay, according to the instructions from the manufacturer. Cells were grown in 96-well plates at a concentration of 1x10^3^ cells/well, and treated with test drugs for 12, 24, 48 or 72 hours. After treatment the level of caspase activity was measured using the Apo-ONE® homogenous caspase-3/7 assay (Promega, Southampton, UK), which employs a pro-fluorescent caspase-3/7 substrate that once activated can be detected using a fluorescence plate reader (excitation: 499 nm, emission: 521 nm).

### Statistical analysis

All experiments were repeated a minimum of three times. Statistical analyses were conducted using GraphPad Prism v4.1 (GraphPad Software, La Jolla, CA, USA) using a two-way Analysis of Variance (ANOVA) with Bonferroni post-test correction. A *P* value <0.05 was considered significant.

## Results

### Eicosanoid production

**Figure 1:**
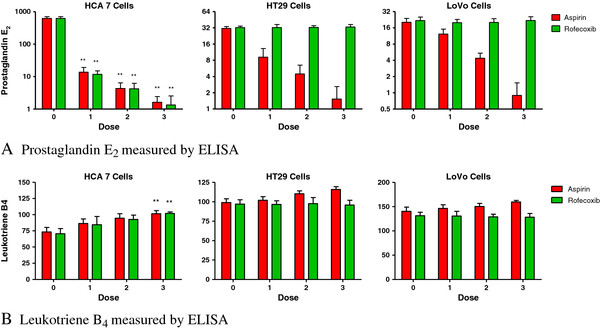
**Effect of aspirin and rofecoxib on (A) prostaglandin E**_**2 **_**and (B) leukotriene B4 secretion by different cell lines.** Cells were cultured without agents (dose 0); with 1 *μ*M rofecoxib, 10 *μ*M aspirin (dose 1); 10 *μ*M rofecoxib, 100 *μ*M aspirin (dose 2); or 100 *μ*M rofecoxib, 1,000 *μ*M aspirin (dose 3). Mean and standard deviations of triplicate cultures shown. (**P <0.001) (** = *P*<0.001).

PGE_2_ production was assayed as a biologically relevant indicator of functional COX-2 activity. Consistent with the level of COX-2 expression in each cell type, HCA7 cells produced the highest concentrations. HT29 cells express an inactive isoform, and LoVo cells do not express COX-2; PGE_2_ release was minimal from these cells. Treatment with aspirin was associated with concentration-dependent reduction in PGE_2_ levels in all cell lines (*P* <0.001). Rofecoxib, as a specific COX-2 inhibitor, reduced PGE_2_ production only in HCA7 cells (Figure 1A).

LTB_4_ was produced by all cells. Aspirin caused a significant increase in production from HCA7 cells (*P* <0.001) and a moderate increase in HT29 and LoVo cells that was not significant. Rofecoxib caused a significant increase in LTB_4_ production in HCA7 cells (*P* <0.001) but did not cause a significant amount of production in other cell lines. (Figure 1B) LTB_4_ was produced by all cells but treatment with aspirin and rofecoxib either increased its production or did not alter its production dependent on cell line.

### Proliferation

We subsequently determined the ability of the test agents to inhibit cellular proliferation. Within 24 hours there was less than 5% reduction in proliferation by aspirin and rofecoxib. Aspirin caused significant inhibition of proliferation only after 72 hours at 1mM dose (*P* <0.05). Rofecoxib did not significantly affect proliferation in any cell line (Table 1). There were no significant differences in the inhibitory capacities between cell lines.

**Table 1:** Effects of aspirin and rofecoxib on cellular proliferation, expressed as reduction relative to control

**24 h**	**Dose**	**HCA7**		**HT29**		**LoVo**	
		**aspirin**	**rofecoxib**	**aspirin**	**rofecoxib**	**aspirin**	**rofecoxib**
	A	2.0 ± 1.3	1.7 ± 1.4	1.2 ± 0.9	1.3 ± 0.9	1.0 ± 0.3	0.9 ± 0.5
	B	2.9 ± 1.9	1.6 ± 0.6	1.9 ± 1.3	1.1 ± 0.2	1.5 ± 0.6	1.7 ± 1.3
	C	2.4 ± 1.1	2.2 ± 0.8	1.7 ± 0.7	1.8 ± 0.8	1.5 ± 0.6	1.8 ± 1.0
48 h	**Dose**	**HCA7**		**HT29**		**LoVo**	
		**aspirin**	**rofecoxib**	**aspirin**	**rofecoxib**	**aspirin**	**rofecoxib**
	A	2.3 ± 1.2	2.3 ± 1.4	2.0 ± 0.9	1.5 ± 0.6	1.8 ± 0.6	1.4 ± 0.8
	B	3.6 ± 1.8	2.5 ± 0.8	2.6 ± 1.7	2.2 ± 1.0	2.7 ± 1.2	2.4 ± 1.3
	C	6.0 ± 2.7	3.8 ± 1.4	3.7 ± 1.7	2.4 ± 1.4	2.7 ± 1.2	2.7 ± 0.9
72 h	**Dose**	**HCA7**		**HT29**		**LoVo**	
		**aspirin**	**rofecoxib**	**aspirin**	**rofecoxib**	**aspirin**	**rofecoxib**
	A	4.6 ± 1.7	2.9 ± 1.6	3.4 ± 1.6	3.0 ± 1.8	2.5 ± 1.0	2.4 ± 0.8
	B	5.0 ± 1.6	3.5 ± 2.0	4.8 ± 2.3	2.9 ± 1.4	2.8 ± 0.9	2.3 ± 1.0
	C	7.7 ± 1.9	4.0 ± 1.6	6.5 ± 2.0	4.1 ± 1.8	5.3 ± 1.8	2.9 ± 1.6

The cells were cultured with 1 *μ*M rofecoxib, 10 *μ*M aspirin (dose A); 10 *μ*M rofecoxib, 100 *μ*M aspirin (dose B); or 100 *μ*M rofecoxib, 1,000 *μ*M aspirin (dose C). Mean and standard deviations are shown.

**Figure 2:**
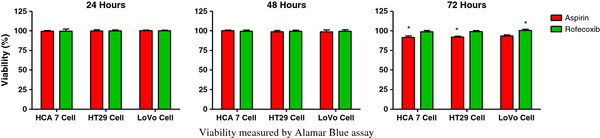
**Effect of 1,000 μM aspirin and 100 μM rofecoxib on viability at 72 hours, relative to control.** Results expressed as mean and standard deviation (* *P* < 0.05).

The assay used to examine proliferation is indirect in that it measures absolute numbers of cells. We therefore tested whether the decreased proliferative potential was due to reduced viability. Aspirin reduced viability by less than 10% in all cell lines at the higher dose used and was only significant at 72 hours at the 1 mM dose (*P* <0.05). Rofecoxib did not affect viability significantly in any cell line tested (Figure 2).

### Apoptosis

**Figure 3:**
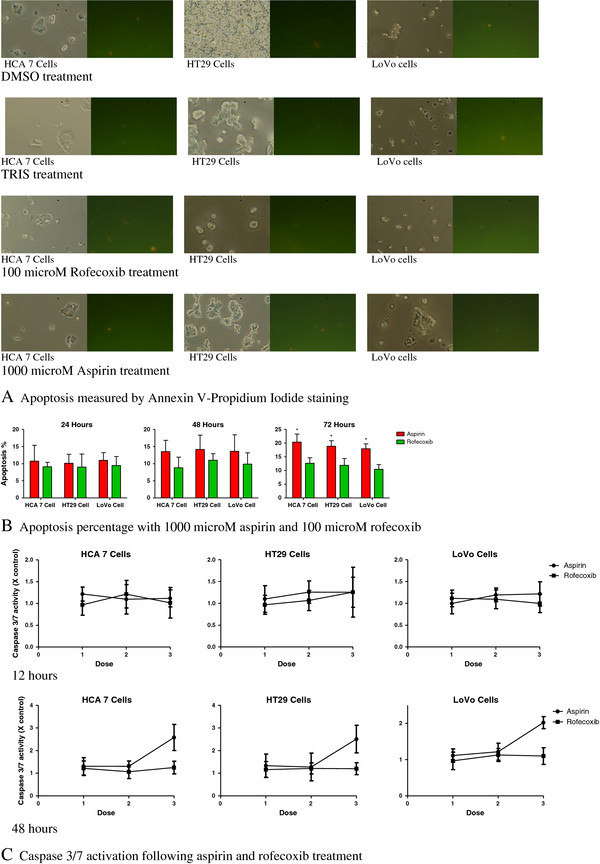
**Effects of 1,000 μM aspirin and 100 μM rofecoxib on apoptosis.** Representative images from light microscopy and immunofluorescence (demonstrating propidium iodide and annexin-V staining) of HCA 7, HT29 and LoVo cells with no agent (control containing DMSO and TRIS), rofecoxib and aspirin (**A**). The apoptosis of cells is shown as percentage at 24, 48 and 72 hours by 1000 μM aspirin, 100 μM rofecoxib. Results are expressed as mean percentage of apoptosis +/− SD (*P <0.05) (**B**). Caspase-3/7 activation relative to control at 12 and 48 hours. Mean and standard deviations of triplicate cultures shown (**C**).

Chemopreventative properties of agents often correlate with the degree of induction of apoptosis, which appears to provide a reliable biomarker for the evaluation of potential novel therapeutic agents. We quantified the number of apoptotic cells using Annexin-V/propidium iodide staining. Annexin-V binds phosphatidyl serine that is externalized to the cell surface with the loss of membrane integrity occurring during the early stages of apoptosis. Propidium iodide differentiates late apoptotic and necrotic cells as it can only permeate cells during these stages (Figure 3A). Aspirin did not induce significant apoptosis for up to 48 hours in all cell lines. Aspirin at 1 mM caused significant apoptosis only at 72 hours of treatment (*P* <0.05), and rofecoxib had no apoptotic effect in all cell lines (Figure 3B).

Caspase induction is the final common pathway in the various apoptotic signaling cascades. It is activated in advance of any morphological changes of apoptosis. Caspase activity was induced to a significant level by aspirin at 1 mM after 48 hours of treatment in all cell lines and its activity declined at 72 hours. Aspirin at lower dose and rofecoxib failed to induce significant caspase activity in all cell lines (Figure 3C).

## Discussion

Aberrant arachidonic acid metabolism is implicated in CRC carcinogenesis [34]. Manipulation of these pathways offers novel therapeutic strategies to prevent or reverse neoplasia. COX and 5-LOX are the two important enzymes involved in the generation of prostaglandins and leukotrienes. In particular, COX-2 expression is upregulated in CRC and NSAIDs may reverse the carcinogenic process by inhibiting this enzyme. Recent studies also have shown that 5-LOX is expressed in colorectal adenocarcinomas and elevated expression of this enzyme appears to correlate with tumor aggressiveness [20], although the exact mechanism remains incompletely understood. The 5-LOX product leukotriene B4 is shown to promote colorectal cancer in an experimental model [35]. It seems likely, however, that COX-2 and 5-LOX represent an integrated system with a common substrate that regulates the proliferative, metastatic and pro-angiogenic potential of cancer cells. Both enzymes induce cell cycle progression and block apoptosis, enhance chemoresistance, and stimulate angiogenesis, with one convergent target on vascular endothelial growth factor (VEGF) expression and release [36].

COX and 5-LOX are frequently co-expressed, and inhibition of a single pathway may shunt arachidonic acid metabolism towards the alternative enzyme. The striking similarities between their biological functions suggest that molecules that equally block both COX-2 and 5-LOX may represent a novel and promising alternative in colon cancer treatment. In support of this mechanism, studies have shown that dual inhibition of COX-2 and 5-LOX have additive anti-cancer effects when compared to inhibition by either enzyme alone [37].

Whereas 5-LOX is universally expressed by all epithelial cancer cell lines COX-2 expression is variable [38]. The proposed shunting mechanism requires the expression of both enzymes. We intended to investigate that this phenomenon of shunting was not due to COX-2 independent process. Therefore, we used three cancer cell lines with differential COX-2 expression and activity to assess the shunting mechanism. HCA7 cells express active COX-2, HT29 cells express an enzymatically inactive variant [31] and LoVo cells do not express COX-2; all express 5-LOX. We found that HCA7 cells produced excess PGE_2_ by overexpressed COX-2, which was significantly reduced following aspirin and rofecoxib treatment. We observed, that in HCA7 cells, aspirin and rofecoxib treatment caused a reciprocal increase in LTB_4_ secretion. These results confirm the shunting hypothesis. In HT29 and LoVo cells with inactive and absent COX-2 expression LTB_4_ secretion was not affected by COX-2 inhibition.

We next wanted to assess the anti-carcinogenic potential of an NSAID. Aspirin treatment did not induce significant anti-carcinogenic effect for up to 48 hours. Only at 72 hours did 1000 μM aspirin cause a significant anti-cancer effect. Rofecoxib exhibited no anti-cancer effect at all times tested. The level of COX-2 expression of the cell did not have any impact on the anti-carcinogenic effects of NSAID. In COX-2 expressing cells, inhibition of COX-2 caused shunting of AA to the 5-LOX pathway resulting in carcinogenic LTB_4_ production. An increase in LTB_4_ antagonizes the anti-carcinogenic effect caused by a reduction in prostaglandin synthesis. In cells with inactive and absent COX-2 expression, COX-2 inhibition is unlikely to affect its growth. These observations suggest at least partially the existence of a shunting mechanism as well as COX-2 independent effects, and may underscore the importance of simultaneous inhibition of leukotriene production. Activity solely targeting the COX enzymes may be insufficient, possibly contributing to the previous conflicting results in this field [39].

The centrality of COX-2 in the anti-neoplastic actions of NSAIDs has also been questioned; for example, studies have demonstrated that replacement of prostaglandins fails to reverse their anti-cancer effects [40]. Additionally, sulindac is a pro-drug that is converted *in vivo* into its active sulfide and sulfone metabolites. Both of these metabolites inhibit colon cancer cell growth although only the sulfide inhibits prostaglandin synthesis. Finally, NSAIDs can induce apoptosis in HCT-15 cells, which lack COX transcripts [41]. All HCT-15 cells lack COX transcript.

## Conclusion

This study confirms the process of shunting of arachidonic acid metabolism between the COX and 5-LOX pathways in the presence of inhibition of one of these enzymes. The shunting mechanism may explain the failure of COX-2 inhibitors to cause significant anti-carcinogenic effect. The observed *in vitro* effects should be reproduced in an animal model to provide evidence for the role of the shunting mechanism *in vivo*. Intervention that inhibits both the COX-2 pathway and the 5-LOX pathway will prove to be an effective anti-carcinogenic agent.

## Abbreviations

AA: arachidonic acid; COX: cyclooxygenase; CRC: colorectal cancer; dsDNA: double-stranded DNA; ELISA: enzyme-linked immunosorbent assay; LTB_4_: leukotriene B_4_; NSAIDs: non-steroidal anti-inflammatory drugs; PGE_2_: prostaglandin E_2_; VEGF: vascular endothelial growth factor; 5-LOX: 5-lipoxygenase.

## Competing interests

The authors declare that they have no competing interests.

## Authors’ contributions

RG contributed in conception, carried out experimental assays, acquisition of data,analysis, interpretation of data and in preparing the manuscript. DJBM involved in statistical analysis and drafting the manuscript. KS contributed in conception, design, experimental assay and interpretation of data and statistical analysis. MW contributed in conception, design and interpretation of the data. AS contribution includes conception, design, in carrying out experimental assay and interpretation of the data. All authors read and approved the final manuscript.
